# TGFα-PE38 enhances cytotoxic T-lymphocyte killing of breast cancer cells

**DOI:** 10.3892/ol.2014.1969

**Published:** 2014-03-12

**Authors:** STEPHEN E. WRIGHT, KATHLEEN A. REWERS-FELKINS, IMELDA QUINLIN, NAZRUL I. CHOWDHURY, JEWEL AHMED, PAUL W. ELDRIDGE, SANJAY K. SRIVASTAVA, IRA PASTAN

**Affiliations:** 1 Women’s Health Research Institute, Department of Internal Medicine, Texas Tech University Health Sciences Center, Amarillo, TX 79106, USA; 2 Department of Microbiology and Immunology, School of Medicine, Texas Tech University Health Sciences Center, Amarillo, TX 79106, USA; 3 Department of Biomedical Sciences, School of Pharmacy, Texas Tech University Health Sciences Center, Amarillo, TX 79106, USA; 4 Harrington Cancer Center, Amarillo, TX 79106, USA; 5 Laboratory of Molecular Biology, Center for Cancer Research, National Cancer Institute, National Institutes of Health, Bethesda, MD 20892-4264, USA

**Keywords:** TGFα-PE38, cytotoxic T lymphocyte, cancer, breast, mucin-1

## Abstract

The aim of the present study was to determine whether the combination of two modalities of immunotherapy, targeting two different tumor antigens, may be feasible and non-toxic, yet enhance the killing of a human breast cancer cell line. The first modality was tumor growth factor α-*Pseudomonas* exotoxin 38 (TGFα-PE38), which specifically targets and kills tumor cells that express the epidermal growth factor receptor. The second modality was mucin-1 (MUC1)-specific cytotoxic T lymphocytes (CTLs), generated by MUC1 stimulation of peripheral blood mononuclear cells, to target the human breast cancer cell line, MCF7. TGFα-PE38 exhibited specific lysis of the MCF7 cells in a concentration- and time-dependent manner. TGFα-PE38 did not kill the normal hematopoietic stem cells or CTLs. Furthermore, TGFα-PE38 was not inhibitory for the growth or differentiation of the normal human hematopoietic stem cells into erythroid and myeloid colonies. In addition, TGFα-PE38 did not inhibit the killing function of CTLs, either when preincubated or co-incubated with CTLs. Finally, therapeutic enhancement was observed, in that TGFα-PE38 and CTLs were additive in the specific lysis of the MCF7 cells. These two modalities of immunotherapy may be beneficial for humans with breast cancer with or without other therapies, including autologous hematopoietic stem cell transplantation, specifically for purging cancer cells from hematopoietic stem cells prior to transplantation.

## Introduction

Therapeutic agents that target tumor specific antigens have the potential to produce effective treatment while minimizing side-effects. Combining therapeutic agents that target more than one tumor antigen may enhance the killing of tumor cells. In addition, a combination of therapeutic agents, targeting multiple antigens, may prevent the escape of tumor cells if one of the antigens is no longer presented. One such tumor antigen that is overexpressed on tumor cells is the epidermal growth factor receptor (EGFR) (1). Targeting may be via the ligand for EGFR, EGF. Fusion of a toxin to a ligand for the receptor produces a specifically targeted cytotoxic agent (1). Receptor-mediated endocytosis internalizes the toxin, which causes cell death (2). One such toxin is *Pseudomonas* exotoxin (PE) (1). Tumor growth factor α (TGFα) is similar in structure to EGF and binds to EGFR at a similar rate (3). Since conjugation of PE to EGF is inefficient, TGFα is often selected as an alternative (1). TGFα-PE38 is cytotoxic to tumor cells *in vitro* (4). Furthermore, a preclinical study in athymic, nude mice demonstrated the effectiveness of TGFα-PE38 in reducing the size of human brain tumor cell lines (5). Based on these studies, TGFα-PE38 was selected as one of the targeted therapies.

The second targeted therapy was that of mucin-1 (MUC1)-stimulated peripheral blood mononuclear cells (PBMCs; M1SMC), which produce cytotoxic T lymphocytes (CTLs) that kill human breast cancer cells *in vitro* (6) and prevent human breast cancer cell tumor development *in vivo* in non-obese diabetic, severe combined immunodeficient (NOD-SCID) mice (7).

## Materials and methods

### Human cells

All human cells were obtained from deceased subjects in accordance with the Texas Tech University Health Sciences Center institutional review board. The study was approved by the ethics committee of Texas Tech University Health Sciences Centre (Amarillo, TX, USA). Frozen human peripheral blood hematopoietic stem cells (HSCs) were obtained from the Bone Marrow Transplant Laboratory of the Harrington Cancer Center (Amarillo, TX, USA) from deceased, anonymous donors. Frozen PBMCs were acquired by apheresis from a deceased, anonymous donor with breast adenocarcinoma.

TGFα-PE38, a gift from Ira Pastan, National Cancer Institute (Bethesda Maryland, USA), was aliquoted and stored at −70°C.

### MUC1 peptides

A single repeat of the MUC1-variable number tandem repeat (VNTR)1 peptide, GSTAPPAHGVTSAPD TRPAP (8), was synthesized by American Peptide Co., Inc. (Sunnyvale, CA, USA).

### Cell culture conditions

Procedures were performed as described previously (6). The PBMCs were not HLA typed, since our previous studies (7,9,10) and other studies (11) have found that cytotoxicity by M1SMC may be non-major histocompatibility complex (MHC)-restricted. Cells were cultured at 2×10^6^ cells/ml in AIM-V^®^ serum-free lymphocyte medium (GIBCO-BRL, Life Technologies Inc., Grand Island, NY, USA) and maintained in a 37°C humidified 5% CO_2_ atmosphere (12). Interleukin (IL)-2 (Cetus Corporation, Berkeley, CA, USA) was added twice per week at 100 IU/ml. The cells were stimulated with MUC1-VNTR1 peptide on days zero and seven, at 1 μg/ml. The cells were harvested on day eight. HSCs were cultured at 2×10^6^ cells/ml in AIM-V^®^.

### Cytotoxicity assays

The MCF7 (HLA-A2) breast cancer cell line was obtained from the American Type Culture Collection (ATCC, Manassas, VA, USA) and cultured as recommended. MCF7 cells express hypoglycosylated mucin (13). This cell line was used as the target cell line in an XTT^®^ assay (Roche Diagnostics Corp., Indianapolis, IN, USA) (14,15) performed according to the manufacturer’s instructions. The TGFα-PE38 or effector cells were tested at a concentration of effector-to-target cell ratio of 1.25. Medium was added in place of effector cells to the spontaneous release control wells. The effector and target cells were incubated together for 18 h, which we had previously found to be superior to 4 h (6), at 37°C and 5% CO_2_. This was performed in triplicate. The percentage of specific lysis (%SL) = [OD(target - medium) − OD(A – B)]/OD(target - medium) ×100, where A represents the experimental (target plus effectors) wells and B represents the wells with a corresponding number of effectors.

### Other assays

Other assays included the Trypan Blue Cell Viability Solution assay (Sigma-Aldrich, St. Louis, MO, USA) (16), the DePsiphler Mitochondrial Potential assay (R&D Systems, Inc., Minneapolis, MN, USA) (17) and the *in situ* chromium uptake assay (18), performed according to the manufacturer’s instructions.

### Progenitor assay

The cells to be tested for progenitor cell content were thawed and washed to remove cryoprotectant. The cells were counted and added to cultures at 5×10^5^ cells/ml in methylcellulose (MethoCult media™; Stem Cell Technologies, Vancouver, BC, Canada) according to the manufacturer’s instructions (19). Cultures were examined after 14 days and scored for the presence of erythroid [burst forming unit-erythroid (BFU-E)] and myeloid (colony-forming unit-granulocytes, monocytes (CFU-GM)] progenitor colonies.

### Statistical analysis

Fisher’s exact test, the χ^2^ test, the Kruskal-Wallis test and the Mann-Whitney signed rank test were used to analyze data. P<0.05 was considered to indicate a statistically significant difference.

## Results

### Concentration- and time-dependent specific lysis of MCF7 cells by TGFα-PE38

**Figure 1:**
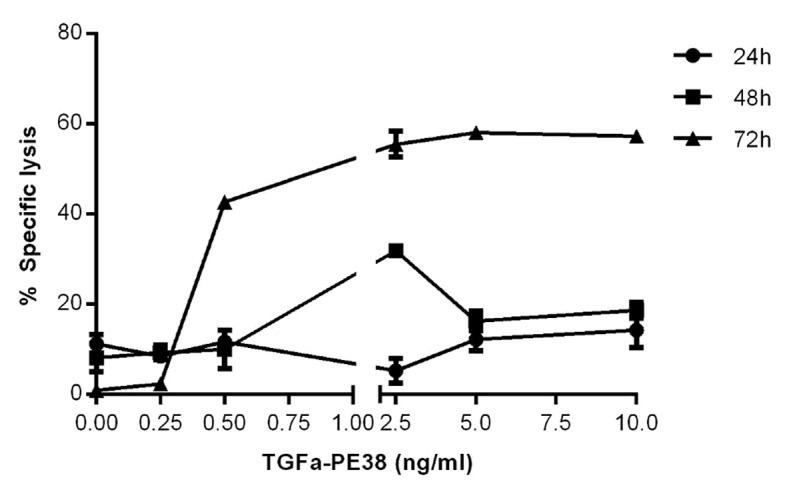
Concentration-dependent specific lysis of MCF7 cells by TGFα-PE38. MCF7 cells were incubated with increasing concentrations of TGFα-PE38 for 24, 48 or 72 h. The percentage of specific lysis (%SL) was determined by the XTT assay. This data is representative of two experiments using the XTT assay. Bars are standard error of the mean (N=3). Error bars are hidden in some of the data points. Other assays, including the Trypan Blue Cell Viability Solution assay, the DePsiphler mitochondrial potential assay and the *in situ* chromium uptake assay, produced similar results, but with a wider range of error. TGF, tumor growth factor; PE, *Pseudomonas* endotoxin.

**Figure 2:**
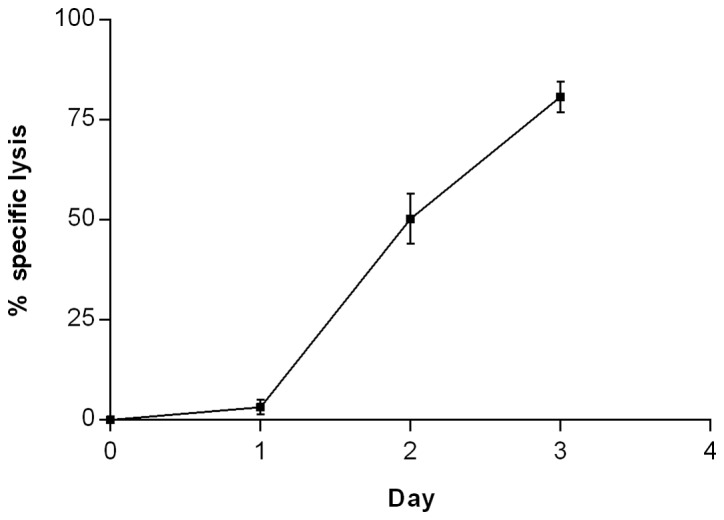
Time-dependent specific lysis of MCF7 cells by TGFα-PE38. MCF7 cells were incubated with 5 ng/ml TGFα-PE38 for 1, 2 or 3 days. The percentage of specific lysis (%SL) was determined by the XTT assay. This data is representative of two experiments using the XTT assay. Bars are standard error of the mean (n=3). Other assays, including the trypan blue viability assay and the *in situ* chromium uptake assay, produced similar results, but with a wider range of error. TGF, tumor growth factor; PE, *Pseudomonas* endotoxin.

Based upon the reported IC_50_ (concentration of immunotoxin causing a 50% reduction in protein synthesis) for MCF7 cells of 1.1 ng/ml when using TGFα-PE38 (4), an analysis of the %SL of the MCF7 cells at a range of TGFα-PE38 concentrations below and above this value were performed to determine the optimum concentration for use in combination with CTLs. The 50% lysis of the MCF7 cells by TGFα-PE38, which was between 0.5 and 2.5 ng/ml at 72 h of incubation (Fig. 1), agreed with the reported IC_50_ data (4). Other assays, including the Trypan Blue Cell Viability Solution assay (16), the DePsiphler Mitochondrial Potential assay (17) and the *in situ* chromium uptake assay (18), produced similar results, but with a wider range of error. The %SL plateaued at 5 ng/ml TGFα-PE38 (Fig. 1) and thus was used for the study of time-dependent specific lysis of the MCF7 cells by TGFα-PE38. There was little (3%) specific lysis of the MCF7 cells by TGFα-PE38 at 24 h, however, this increased exponentially to 50 and 81% at 48 and 72 h, respectively (Fig. 2).

### Effect of TGFα-PE38 on HSC viability

**Figure 3:**
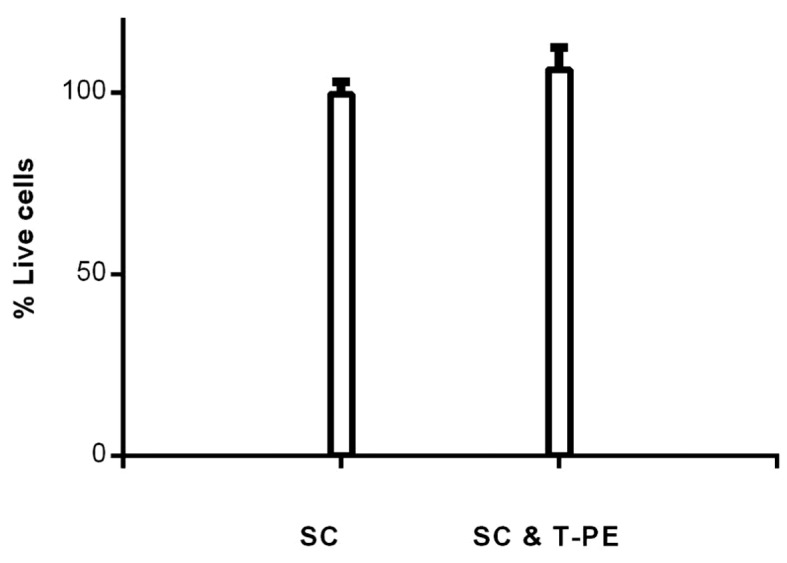
Effect of TGFα-PE38 (T-PE) on hematopoietic stem cell (HSC) viability. HSC were incubated with 5 ng/ml T-PE for five days. n=4 without T-PE and n=9 with T-PE. The percentage of remaining live cells was determined by the XTT assay. Bars are standard error of the mean. TGF, tumor growth factor; PE, *Pseudomonas* endotoxin.

To determine if TGFα-PE38 was non-toxic in normal cells, frozen human peripheral blood HSCs were thawed and incubated with or without 5 ng/ml TGFα-PE38 for five days. The five-day incubation time was selected, as we had shown that the majority of MCF7 cells were killed by TGFα-PE38 at 72 h. The percentage of remaining live HSCs was similar with or without TGFα-PE38 (106 and 100%, respectively; Fig. 3).

### Effect of TGFα-PE38 and/or CTLs on HSC growth and differentiation

**Figure 4:**
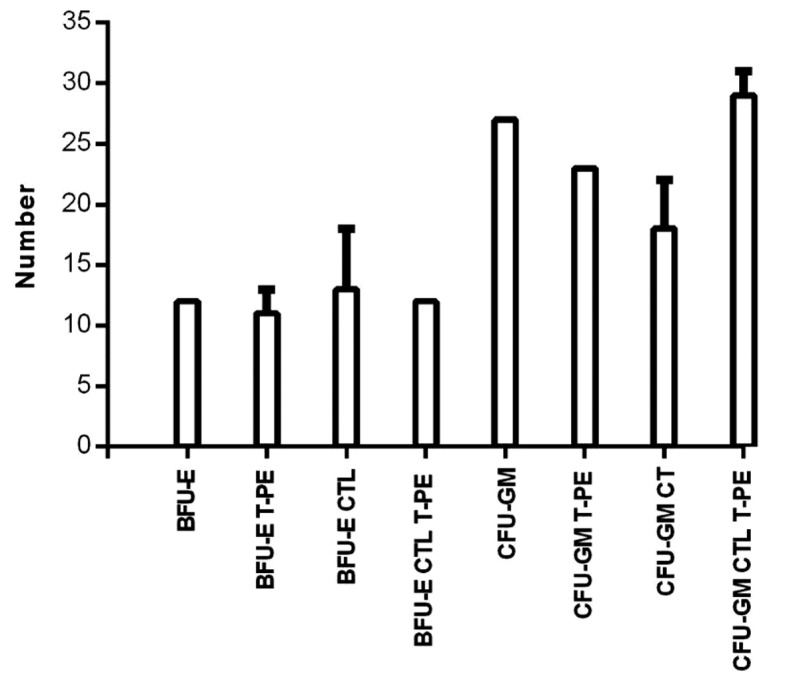
Effect of TGFα-PE38 (T-PE) on hematopoietic stem cell (HSC) erythroid [burst forming unit-erythroid (BFU-E)] and myeloid [colony-forming unit-granulocytes, monocytes (CFU-GM)] progenitor colonies. HSCs were incubated with or without 5 ng/ml T-PE and/or CTLs at an effector-to-target ratio of 1.25 for 14 days. The number of BFU-E and CFU-GM were determined by a methylcellulose assay. There was no statistical difference in any group of BFU-E or CFU-GM compared with the other BFU-E or CFU-GM groups, respectively (Kruskal-Wallis test). Bars are standard error of the mean (n=2). Error bars are hidden in some of the data points. TGF, tumor growth factor; PE, *Pseudomonas* endotoxin; CTL, cytotoxic T lymphocytes.

To determine if TGFα-PE38 and/or CTLs were inhibitory for growth and differentiation of normal cells, frozen human peripheral blood HSCs were thawed and incubated with or without 5 ng/ml TGFα-PE38 and/or CTLs for 14 days. The 14-day incubation time was selected since this is the time required to see macroscopic colonies in order to assay for BFU-E and CFU-GM (19). The mean number of BFU-E and CFU-GM were not significantly different with or without TGFα-PE38 (BFU-E with, 11, and without, 12; and CFU-GM with, 23, and without, 27) or for CTLs with or without TGFα-PE38 (BFU-E with, 12, and without, 13; and CFU-GM with, 29, and without, 18) (Fig. 4).

### Effect of TGFα-PE38 on CTL cell viability

**Figure 5:**
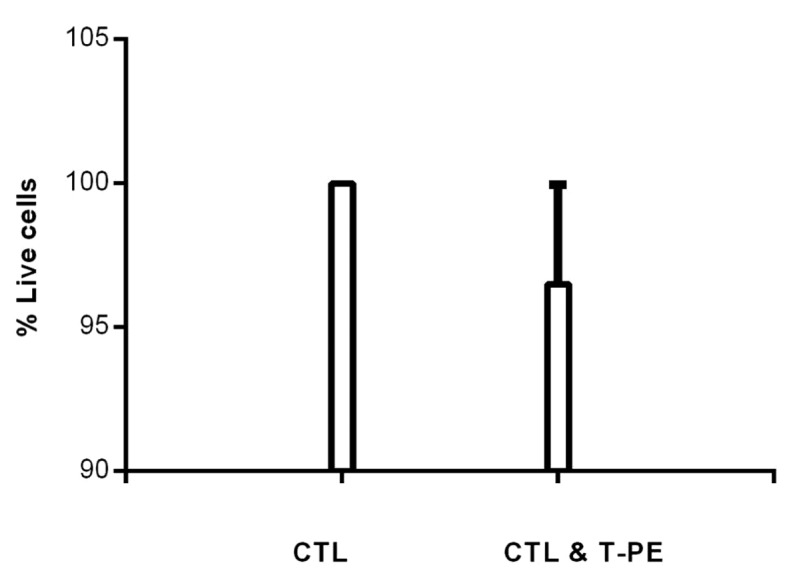
Effect of TGFα-PE38 (T-PE) on CTL cell viability. CTLs were incubated with 5 ng/ml T-PE for three days. The percentage of remaining live cells was determined by the XTT assay. CTLs incubated without T-PE versus with T-PE, P=0.87 (χ^2^ test). Bars are standard error of the mean (n=2). CTLs alone was set to 100%, so the error bar is not shown. TGF, tumor growth factor; PE, *Pseudomonas* endotoxin; CTL, cytotoxic T lymphocytes.

To determine if TGFα-PE38 was non-toxic for CTLs, CTLs were incubated with or without 5 ng/ml TGFα-PE38 for three days. The three-day incubation time was selected, as we had shown that the majority of the MCF7 cells were killed by TGFα-PE38 at 72 h and that the lytic function of CTLs was reduced with time in culture (6). The percentage of remaining live CTLs was similar with or without TGFα-PE38 (97 and 100%, respectively; Fig. 5).

### Effect of TGFα-PE38 on the killing function of CTLs

**Figure 6:**
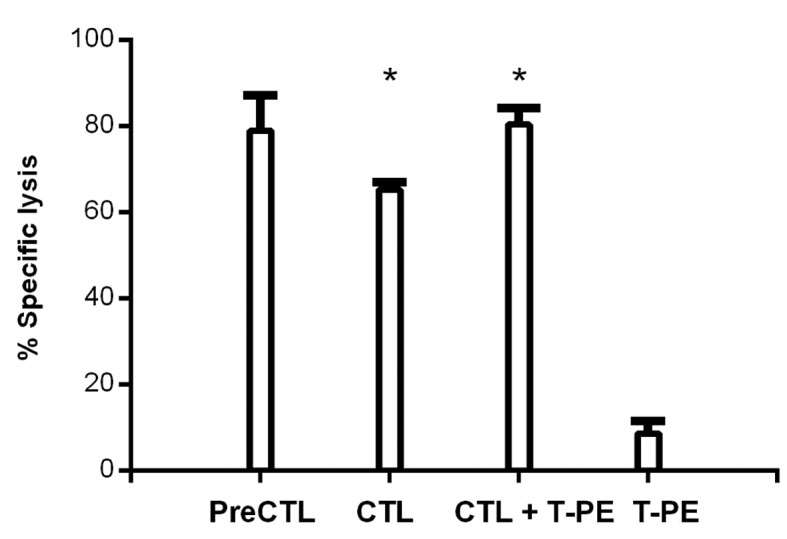
Specific lysis of MCF7 by CTLs, preincubated (PreCTLs) or not (CTL) with TGFα-PE38 (T-PE), and CTLs co-incubated with T-PE (CTL + T-PE) or not. MCF7 cells were incubated with CTLs preincubated with 0.25 ng/ml T-PE for one day (PreCTL) or not (CTL) at an effector (CTL)-to-target (MCF7) ratio (E:T) of 1.25 for one day. The third group was CTLs at an E:T of 1.25 co-incubated with or without 0.25 ng/ml T-PE for one day (CTL + T-PE). The percentage of specific lysis (%SL) was determined by the XTT assay. Preincubation of CTLs with T-PE (Pre CTL) vs. CTL, P=0.48 (NS; Mann-Whitney Rank Sum test). CTLs co-incubated with T-PE (CTL + T-PE) vs. CTL, ^*^P=0.01. Pre CTL vs. CTL + T-PE, P=0.22 (NS). Results were similar for E:T ratios of 0.625 and 2.5. Bars are standard error of the mean (n=3). TGF, tumor growth factor; PE, *Pseudomonas* endotoxin; CTL, cytotoxic T lymphocytes; NS, not significant.

To determine if TGFα-PE38 inhibited the killing function of CTLs, CTLs were incubated with or without TGFα-PE38 for one day. The one-day preincubation time was selected, as the lytic function of CTLs is reduced with time in culture (6). The %SL of the MCF7 cells by CTLs was not inferior following preincubation with TGFα-PE38 (79%) versus without TGFα-PE38 (65%) (Fig. 6). To determine if TGFα-PE38 enhanced the specific lysis of the MCF7 cells by CTLs, TGFα-PE38 was co-incubated with CTLs in a one-day specific lysis assay of the MCF7 cells. The one-day incubation time was selected, as this is the standard time for the MCF7 specific lysis assay (6). The concentration of TGFα-PE38 was lowered to obtain a specific lysis of the MCF7 cells of 9%, so that an additive effect of CTLs could be observed. Co-incubation with CTLs and TGFα-PE38 produced additive effects in the specific lysis of the MCF7 cells (80%) (Fig. 6). This result was significantly different (P=0.01) to CTLs alone (65%). The specific lysis of the MCF7 cells following co-incubation with CTLs and TGFα-PE38 (80%) was not significantly different (P=0.22) to CTLs preincubated with TGFα-PE38 (79%) (Fig. 6).

## Discussion

We aimed to affirm or refute whether the combination of two modalities of immunotherapy, targeting two different tumor antigens, may be feasible and non-toxic, yet enhance killing of a human breast cancer cell line. The first target selected was EGFR, since it is present on multiple types of tumors (1). Since TGFα is similar in structure to EGF and binds to EGFR at a similar rate as EGF (3), it was selected as the ligand for EGFR. Additionally, since fusion of a toxin to a ligand for the receptor produces a specifically targeted cytotoxic agent (1), a ligand, TGFα, fused to a toxin, PE, was used (1). TGFα-PE38 has been shown to be cytotoxic to tumor cells *in vitro* (4). In addition, TGFα-PE38 reduces the size of human brain tumor cell lines in athymic, nude mice (5). As predicted from these previous studies, TGFα-PE38 was shown to lyse a human breast cancer cell line, MCF7, in a concentration- and time-dependent manner. In order for a product to be used in humans it must be non-toxic for normal cells. Therefore, TGFα-PE38 was evaluated for toxicity against normal cells. It was non-toxic for normal cells, specifically for frozen human peripheral blood HSCs. Furthermore, TGFα-PE38 did not inhibit the function of peripheral blood HSCs. The second targeted therapy was M1SMC, which produce CTLs. We previously demonstrated that these kill human breast cancer cells *in vitro* (6), and prevent human breast cancer cell tumor development *in vivo* in NOD-SCID mice (7). TGFα-PE38 was not only non-toxic to CTLs, but it also did not inhibit the specific lysis of a human breast cancer cell line by CTLs, either as a preincubation or in co-incubation. Instead, TGFα-PE38 enhanced the specific lysis of a human breast cancer cell line by CTLs. These results support the enhancement of tumor cell killing using two targets for immunotherapy, as we have previously shown with two different targets for developing CTLs (20).

In summary, the targeting of two tumor antigens by two different immunotherapeutic modalities was shown to be feasible, non-toxic and superior to either of the individual modalities in the specific lysis of a human breast cancer cell line. The combination of these two modalities of therapy, targeting two different tumor antigens, may be of use in humans in preventing recurrence of breast cancer following autologous hematopoietic stem cell transplantation (21,22), by purging the contaminating breast cancer cells, which are associated with recurrence (23), from the hematopoietic stem cells. In addition, these two modalities of immunotherapy may be of benefit *in vivo* for humans with breast cancer with or without other therapies, including following autologous hematopoietic stem cell transplantation.
